# Treatment of cancer cells with Lapatinib negatively regulates general translation and induces stress granules formation

**DOI:** 10.1371/journal.pone.0231894

**Published:** 2020-05-04

**Authors:** Pauline Adjibade, Bryan Simoneau, Nassim Ledoux, William-Naud Gauthier, Melisse Nkurunziza, Edouard W. Khandjian, Rachid Mazroui

**Affiliations:** 1 Département de Biologie Moléculaire, Biochimie Médicale et Pathologie, Centre de Recherche en Cancérologie, Centre de Recherche du CHU de Québec, Faculté de Médecine, Université Laval, Québec, Parti Québécois, Canada; 2 Département de Psychiatrie et de Neurosciences, Centre de Recherche, Institut Universitaire en Santé mentale de Québec, Faculté de Médecine, Université Laval, Québec, Parti Québécois, Canada; Wayne State University, UNITED STATES

## Abstract

Stress granules (SG) are cytoplasmic RNA granules that form during various types of stress known to inhibit general translation, including oxidative stress, hypoxia, endoplasmic reticulum stress (ER), ionizing radiations or viral infection. Induction of these SG promotes cell survival in part through sequestration of proapoptotic molecules, resulting in the inactivation of cell death pathways. SG also form in cancer cells, but studies investigating their formation upon treatment with chemotherapeutics are very limited. Here we identified Lapatinib (Tykerb / Tyverb^®^), a tyrosine kinase inhibitor used for the treatment of breast cancers as a new inducer of SG in breast cancer cells. Lapatinib-induced SG formation correlates with the inhibition of general translation initiation which involves the phosphorylation of the translation initiation factor eIF2α through the kinase PERK. Disrupting PERK-SG formation by PERK depletion experiments sensitizes resistant breast cancer cells to Lapatinib. This study further supports the assumption that treatment with anticancer drugs activates the SG pathway, which may constitute an intrinsic stress response used by cancer cells to resist treatment.

## Introduction

Stress granules (also referred as cytoplasmic phase transition or droplets) are RNA cytoplasmic foci that emerge as a result of accumulation of either untranslated mRNAs or deficient translation initiation complexes [1–3] when general translation initiation is blocked. This occurs during various translational stresses known to inhibit general translation including treatment with genotoxic drugs inducers of oxidative and ER stress, exposure to hypoxia, and treatment with either heat shock or radiation [4,5].

During translational stress, the initiation of general translation is blocked mainly due to the phosphorylation of the translation initiation factor eIF2α [6,7]. eIF2α is phosphorylated by four specific stress kinases. GCN2 (general control nonderepressible 2) phosphorylates eIF2α during amino acid deprivation [8] and PKR (Protein kinase R) is responsible for eIF2α phosphorylation during viral infection [9]. While HRI (heme-regulated inhibitor kinase) is activated and phosphorylates eIF2α in response to oxidative stress, heme deficiency, and proteasome inhibition [10], PERK (PKR-like endoplasmic reticulum kinase) phosphorylates eIF2α during endoplasmic reticulum stress [7,11]. Once phosphorylated, eIF2α induces stalling of translation initiation complexes in an inactive form whose accumulation results on SG formation [12].

Super-resolution fluorescence microscopy analysis of SG combined with biochemical purifications of their components suggest that SG consist of a stable core that can be biochemically purified, surrounded by a shell with highly dynamic components [13]. Among other components, SG consist of mRNA, translation machinery including initiation factors and small ribosomal subunits, RNA binding proteins with disorganised SG-nucleating motifs (TIA-1, FMRP, G3BP), and signaling molecules (e.g., and RACK1) involved in cell death [4]. Sequestration of specific signaling molecules into SG has been reported as a potential SG-based survival mechanism [14,15]. SG can also assist the expression of key survival proteins by preventing the degradation of encoded mRNAs, which may thus promote cell survival [16,17]. Although SG formation was implicated in cell survival, limited reports have assessed their formation during therapeutic stress induced by either chemo- or radiotherapy and the role of this formation in cancer cells resistance to treatment.

Lapatinib (Tykerb /Tyverb) is a dual tyrosine kinase inhibitor which interrupts the HER2/neu receptor (human EGFR type 2) and epidermal growth factor receptor (EGFR) signaling and it is used to treat HER2-positive breast cancers [18,19]. However, patients often experience progression due to both primary unresponsiveness and inquired Lapatinib resistance [20,21]. Here, we found that Lapatinib treatment induces SG formation in cancer cells including those derived from breast cancer. This SG formation requires the activation of PERK kinase phosphorylating eIF2α, causing the inhibition of general translation. PERK depletion decreases cell survival following treatment with Lapatinib, potentially by inhibiting SG formation. These data further support the assumption that SG formation induced by chemotherapeutic agents might be a more general mechanism with potential significance in chemoresistance.

## Material and methods

### Cell lines and cell culture

Human breast cancer cell lines T47D and MCF-7 were cultured in RPMI-1640 medium (wisent) supplemented with 5% fetal bovine serum (Wisent), penicillin and streptomycin (wisent). All the cells were maintained in an environment with a humidified incubator with 5% CO2 at 37°C. U2OS were obtained from Dr. A Fradet- Turcotte (Université Laval).

### Antibodies and reagents

Anti-FMRP, anti-FXR1 and anti-G3BP antibodies have been previously described [22]. Anti-eIF4GI, phospho-specific anti-eIF2α, the pan anti-eIF2α, anti-4EBP1 and anti-phospho-4EBP1 were purchased from Cell Signaling Technology (Beverly, MA, USA). Anti-DDX3, -PERK, -GCN2 and -α-tubulin antibodies were purchased from Abcam. Anti-m [6] A antibody was obtained from Synaptic Systems (Germany). Anti-HRI antibodies were purchased from Santa Cruz Biotech (Santa Cruz, CA, USA). Anti-puromycin antibody was obtained from EMD Millipore (Merck, Germany). Anti-Vinculin was provided by Dr. M-E Huot (Université Laval). Anti-Dcp1a and anti-LSM14A have been described previously [23,24].

### Drug treatment

Lapatinib was purchased from Selleck Chemicals and dissolved in DMSO as a 10 mM stock solution, aliquoted and stored at −80 °C. Cycloheximide and puromycin were purchased from Sigma (St Louis, MO) and dissolved in water as a 10 mg/mL and 25 mg/mL stock solutions, respectively, aliquoted and stored at −20°C. ISRIB was purchased from Sigma (St Louis, MO). For drugs treatment, cells were plated to reach a confluency of ~80% the day of the treatment. The media was changed with fresh media two hours before treatment.

### Confocal immunofluorescence

For immunofluorescence, cells were fixed with 3,7% paraformaldehyde and permeabilized. The sample were blocked with 1% BSA and incubated with primary antibodies diluted in 0.1% Tween-20 in PBS (PBST). Cells were then incubated with secondary antibodies coupled to Alexa Fluor 488/ 594. Sample were visualized using the LSM 700 laser scanning confocal microscope (Zeiss), equipped with a Zen software for image acquisition and processing.

### Polyribosomal profiles

T47D grown in 100-mm tissue culture (~ 80% confluence), were treated with 20 μM Lapatinib, then lysed in polyribosomal buffer (20 mM Tris, pH 7.4, 150 mM NaCl, 1.25 mM MgCl2, 8 U/ml RNase inhibitor [Invitrogen], protease inhibitor cocktail [Complete; Roche], 1 mM DTT, and Nonidet P-40 at a final concentration of 1%). Extracts were clarified by centrifugation and the resulting cytoplasmic extracts were loaded on 15–55% (w/v) linear sucrose gradient for sedimentation by ultracentrifugation. Continuous monitoring of absorbance at 254 nm was performed to obtain the polyribosomal profiles.

### siRNA and shRNA experiments

siRNAs were purchased from Dharmacon (Lafayette, CO). siRNA transfections were performed, using Hiperfect reagent (Qiagen). Briefly, cells were plated to reach a confluency of ~50% the day of the transfection. For a 6-well plate, annealed duplexes were used at a final concentration of 10 nM. Forty-eight hours post-transfection, cells were treated with siRNA (5 nM) for an additional forty-eight hours. Cells were then either fixed and processed for immunofluorescence or harvested for protein extraction.

ShRNA-mediated depletion of PERK was obtained using lentiviral shRNA. PERK shRNA was generated by ligation of oligonucleotides into the *Age*I and *Eco*RI restriction sites of pLKO.1 (Addgene plasmid #8453). Lentiviral-shRNA particles were generated by transfecting HEK 293T cells with 12 μg of pLJM1 vector containing the shRNA, 6 μg of psPAX2 packaging plasmid (Addgene plasmid #12260) and 2 μg pMD2.G envelope plasmid (Addgene plasmid #12259). Medium was changed 16 h after transfection and lentiviral particles were harvested 24 h later. Viral supernatant was filtered through 0.45 μm filters and supplemented with 8 μg/ml polybrene (Sigma). Supernatant was added to the cells for 24 h before the start of puromycin selection.

The sequences of siRNAs/shRNA used in this study are:

siPERK#1: sense sequence: 5′- GGC AAU GAG AAG UGG AAU U -3′

shPERK#2: sense sequence: 5′- GCU GAA AGA UGA AAG CAC A -3’

### Cell viability assay

Cell viability was determined using the MTT assay. Briefly, cells were seeded at 10 000 cells/well in 96-well plates. Cells were cultured overnight and then treated with 20 μM Lapatinib for 24h. At the end of the treatment, cells were washed with PBS and complete media was added. Two hours before the end of treatment, MTT solvent was added to each of the wells. The absorbance of each well was measured at 560 nm.

## Results

### Lapatinib treatment induce stress granules formation

**Fig 1:**
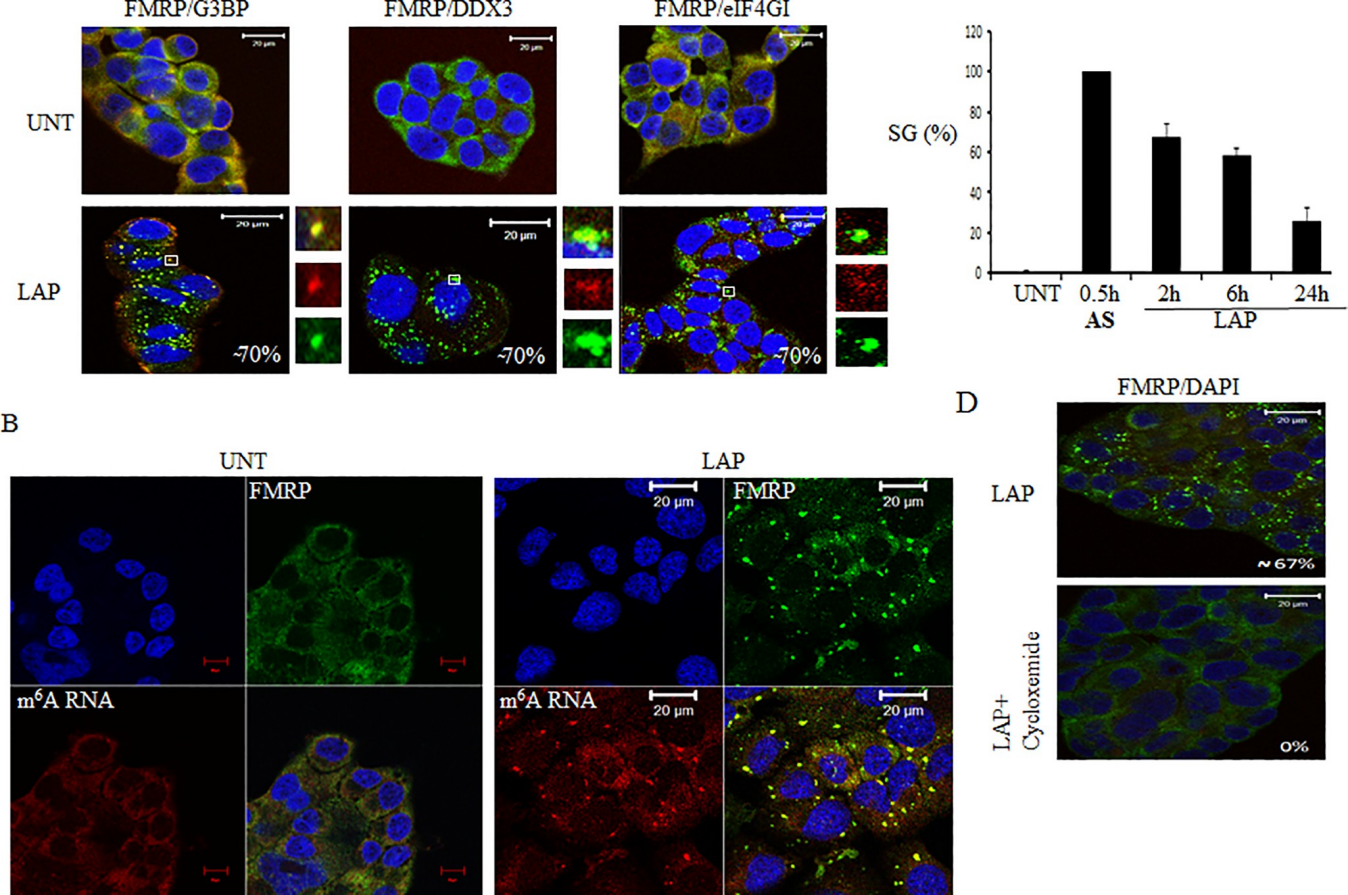
LAP induces SG in T47D. (A) T47D were treated with LAP (20 μM) for two hours. Cells were fixed, permeabilized and processed for immunofluorescence using antibodies against different SG markers (FMRP, G3BP, DDX3, eIF4GI). DAPI is used as a marker for nuclei. Scale bars are shown. The indicated percentage of SG-positive cells (>3 granules / cell) representing more than 1000 cells of five independent experiments. Arsenite (0.5 mM) treatment was used as a control of SG formation. (B) T47D were treated with LAP for two hours. Cells were fixed, permeabilized and processed for immunofluorescence using specific antibodies against the SG marker FMRP and m^6^A. DAPI is used as a marker for nuclei. (C) T47D were treated with LAP, collected at the indicated time points and analysed for SG formation as above. The percentage of SG was calculated as in 1A. (D) T47D were treated with LAP (20 μM) alone or with cycloheximide (100 μg / ml) for two hours, fixed, permeabilized and processed for immunofluorescence using anti-FMRP antibodies to detect SG. DAPI is used as a marker for nuclei. The percentage of SG-positive cells is indicated.

We have previously shown that treatment of cancer cells with sorafenib, a tyrosine kinase inhibitor used to treat hepatocarcinoma induces the formation of SG [25]. Here, we tested if Lapatinib (Lap), the chemotherapeutic that targets breast specific tyrosine kinases, induces SG. We treated breast cancer cell lines, with various doses of Lap and performed immunofluorescence using several antibodies specific to SG markers. Our dose-response experiments show that the maximal SG formation was achieved using 20 μM Lap, which was selected for the rest of our study. As shown in Fig 1A, Lap treatment induces SG formation in ~70% T47D after 2 h of treatment. Lap-induced SG contain classical SG components including FMRP, G3BP, DDX3 and eIF4GI (Fig 1A). N^6^-methyladenosine (m^6^A) is a specific methylation that occurs at specific adenosine residues in a large variety of mRNAs [26]. m^6^A antibodies have been recently used in immunofluorescence experiments, detecting methylated RNA into SG induced by arsenite treatment [27]. Our localisation studies using m^6^A antibodies detect a strong RNA signal in SG indicating that Lap-SG contain RNA (Fig 1B). In addition to SG, RNA is also found in P-bodies, cytoplasmic RNA granules that are constitutively present in unstressed cells [28]. P-bodies are highly dynamics whose number can increase [29], or decrease [23,30] following stress. Using dcp1a and LSM14A, the two classic P-bodies markers, we could not obtain a good staining in T47D. We thus assessed P-bodies formation in U2OS, a cell line that is routinely used for such studies, and which also form SG in response to Lap treatment (S2 Data). Our immunofluorescence experiments show that Lap treatment does not significantly affect P-bodies formation (S2 Data). As previously reported [29], P-bodies are found at close proximity with Lap-induced SG, indicating that they communicate with each other. To monitor the dynamics of SG, we performed time course experiments showing that the maximal SG formation lasted over 6 h, before decreasing after 24 h of Lap treatment, indicating that Lap-induced SG are transient (Fig 1C). Lap-induced SG have also similar dynamic characteristic than classic SG as they are prevented by cycloheximide (Fig 1D); an antibiotic that it is thought to prevent SG formation by blocking the release of mRNPs from translating ribosomes [31,32]. SG formation triggered by Lap treatment is not restricted to T47D as they do form also in the breast cancer MCF-7 cell line, although with less efficiency (S1 Data). However, no SG formation was observed in MDA-MB-231 or Hs578T breast cancer cell lines (S1 Data), indicating that SG formation induced by Lap in breast cancer is cell-type specific.

### Lap treatment activates PERK-phosphorylation of eIF2α (PeIF2α) pathway, and inhibits general translation

**Fig 2:**
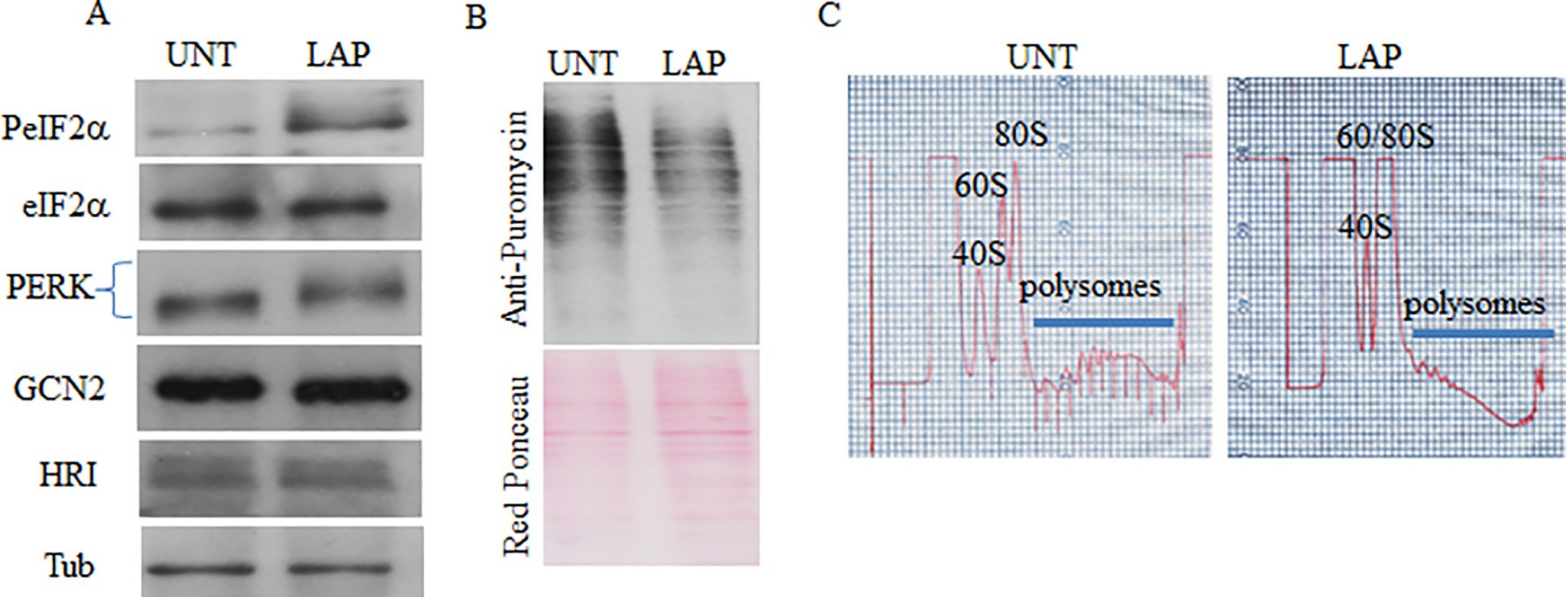
Formation of SG that occurs during Lap treatment correlates with the activation of PERK-PeIF2α pathway and inhibition of general translation. (A-C) T47D were treated with LAP (20 μM) for two hours. (A) Protein extracts were prepared and their content analysed by western blot using antibodies specific to the indicated proteins. Tubulin (Tub) and eIF2α serve as loading controls. (B) Five minutes before the end of the treatment, puromycin (50 μg / ml) was added. Cells were collected and protein content was analyzed by western blot for puromycin incorporation into nascent polypeptide chains using anti-puromycin antibodies (top panel). Red ponceau staining shows equal protein loading (bottom panel). (C) Cytoplasmic extracts were prepared and fractionated onto 15–55% sucrose gradients and the polyribosomes profiles were recorded by measuring the OD_254_. Positions of 40S, 60S, monosomes and polysomes are indicated.

It was previously shown that treatment of BT474 and MCF7 cells with a combination of Lap and obatoclax induces an ER stress response as it increases BiP/GRP78 and IRE1 expression leading to an enhanced phosphorylation of PERK [33]. We thus tested if Lap activates PERK-PeIF2α pathway leading to SG formation. First, we confirmed that Lap treatment activates PERK as assessed by the retarded migration of PERK on a SDS-PAGE indicating its hyper-phosphorylation (Fig 2A). Control western blot experiments show no retarded migration of either HRI or GCN2 activation on a SDS-PAGE, excluding a significant activation of either protein following Lap treatment (Fig 2A). These western blot analyses using antibodies specific to PeIF2α also show that Lap treatment induces phosphorylation of eIF2α. This phosphorylation of eIF2α correlates with an inhibition of general translation as observed by the puromycylation assay [25] showing a significant reduction of puromycin incorporation during Lap treatment (Fig 2B). This result was confirmed by polyribosomal profiles analysis showing a significant loss of polyribosomes, with a concomitant increase in the monosome peaks attesting an inhibition of translation, probably at its initiation phase following Lap treatment (Fig 2C). Together, these results showed that Lap treatment of T47D activates PERK-PeIF2α pathway resulting in an inhibition of translation initiation.

### Lap-induced SG require the phosphorylation of eIF2α mediated by PERK

**Fig 3:**
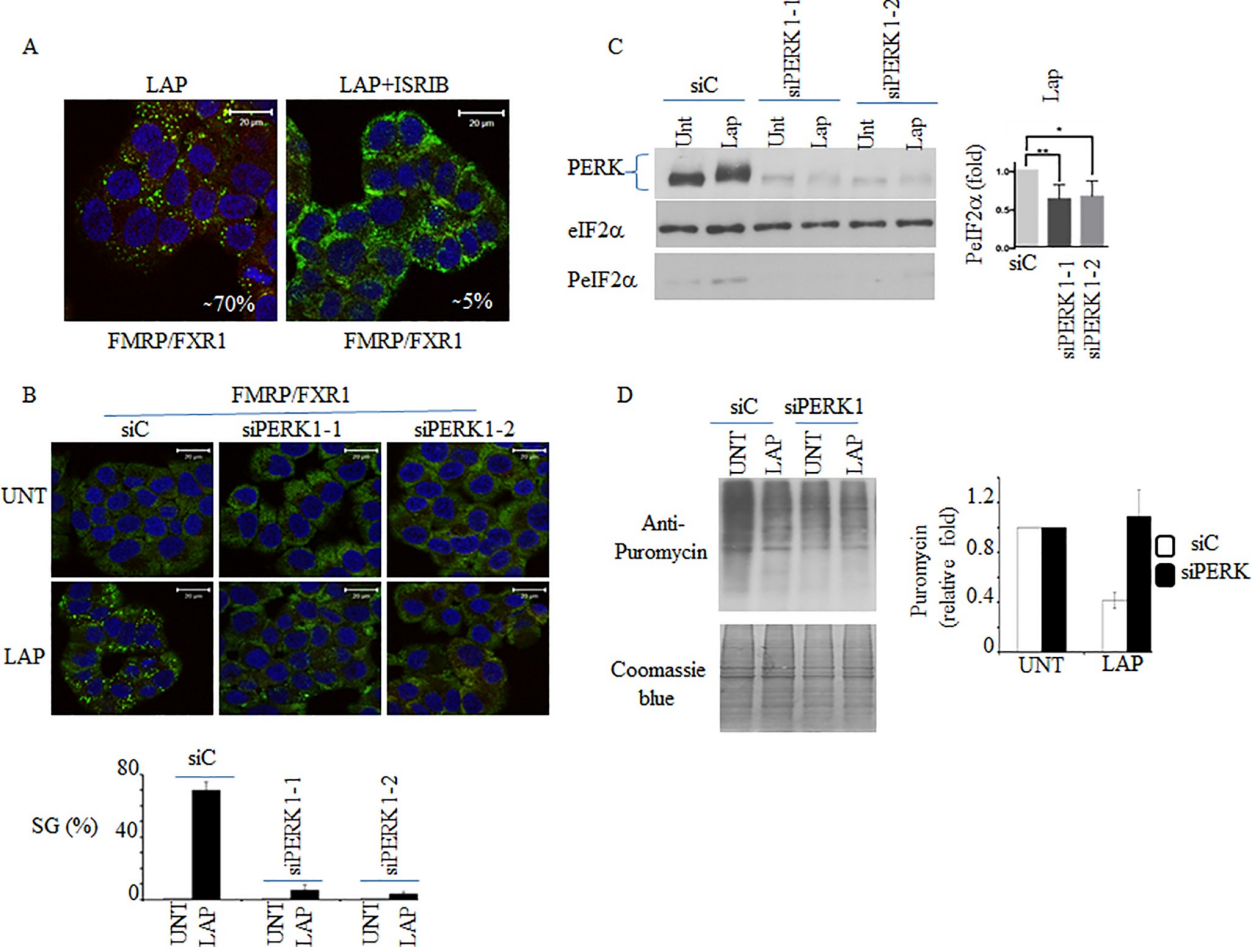
PERK is required for SG formation during LAP treatment. (A) T47D were treated with LAP (20 μM) or with LAP and ISRIB (100 nM) for two hours. Cells were processed for immunofluorescence as above using anti-FMRP and anti-FXR1 antibodies. DAPI stains nuclei. (B-D) T47D were treated with two specific PERK siRNAs and then incubated with LAP (20 μM) for two hours. (B) Cells were fixed and SG were visualised by immunofluorescence using anti-FMRP and anti-FXR1 antibodies. DAPI is used as a marker for nuclei. (C) Cells were collected, and protein content was analyzed by western blot for PERK and PeIF2α. eIF2α serves as loading control. PERK level was estimated by densitometry quantification of the corresponding signal and then normalized to eIF2α. These quantifications revealed a reproducible 70 to 75% PERK depletion. (D) Five minutes before the end of the LAP treatment (20 μM), puromycin (50 μg / ml) was added. Cells were collected and protein content was analyzed by western blot for puromycin incorporation into nascent polypeptide chains using anti-puromycin antibodies (top panel). Coomassie Blue staining shows equal protein loading (bottom panel). Puromycin signals were estimated by densitometry quantification and then normalized to total protein loading assessed by coomassie blue staining and reported as a percentage of the untreated cells.

We then tested if this PERK-PeIF2α stress pathway is responsible for SG formation in Lap-treated cells. First, we assessed the role of eIF2α phosphorylation using ISRIB, a chemical compound that prevents formation of SG that are triggered by eIF2α phosphorylation [34]. We observed that incubation with ISRIB indeed blocked SG formation induced by Lap treatment (Fig 3A) supporting a major role of phosphorylation of eIF2α in triggering SG formation during Lap treatment. In addition of the phosphorylation of eIF2α-mediated SG formation classic pathway, recent studies described a non-canonical pathway of SG formation that relies on the inactivation of the translation initiation eIF4E-eIF4GI complexes through dephosphorylation of 4EBP1 [35]. We observed that Lap treatment also reduces phosphorylation of 4EBP1, albeit modestly, as assessed by western blot using specific antibodies (S3 Data). Together, these results suggest that Lap treatment may also activate the non-classical 4EBP1 dephosphorylation pathway of SG formation, which however requires phosphorylation of eIF2α to occur.

We further assessed the role of the phosphorylation of eIF2α in SG formation using mouse embryonic fibroblasts (MEFs) having an altered PeIF2α pathway [25]. Control experiments show that Lap induces SG in about 15% of wild type MEFs (S4 Data). This SG formation was completely prevented in MEFs expressing S51A mutant eIF2α that cannot be phosphorylated. This result supports that phosphorylation of eIF2α is a key trigger of SG formation during Lap treatment.

Our data described in Fig 2A suggest that PERK is the main eIF2α-phosphorylating kinase that drives SG formation during Lap treatment. We then assessed this possibility using both MEFs and T47D. Our Immunofluorescence experiments show that less than 1% of MEFs lacking PERK are positive for SG in response to Lap treatment, as compared to 15% of wild type MEFs (S4 Data) consolidating the role of PERK in SG formation induced in MEFs upon Lap treatment. PERK knock-down in T47D also prevents significantly SG formation (Fig 3B). Control experiments show that the suppression of SG upon PERK depletion correlates with a decrease in eIF2α phosphorylation (Fig 3C) and a partial rescue of translation inhibition, as compared to mock-depleted cells (Fig 3D). Collectively, these data confirm PERK as a major eIF2α phosphorylating kinase that drives SG formation in cancer cells treated with Lap.

### Implication of PERK-SG formation pathway in Lap resistance in T47D

**Fig 4:**
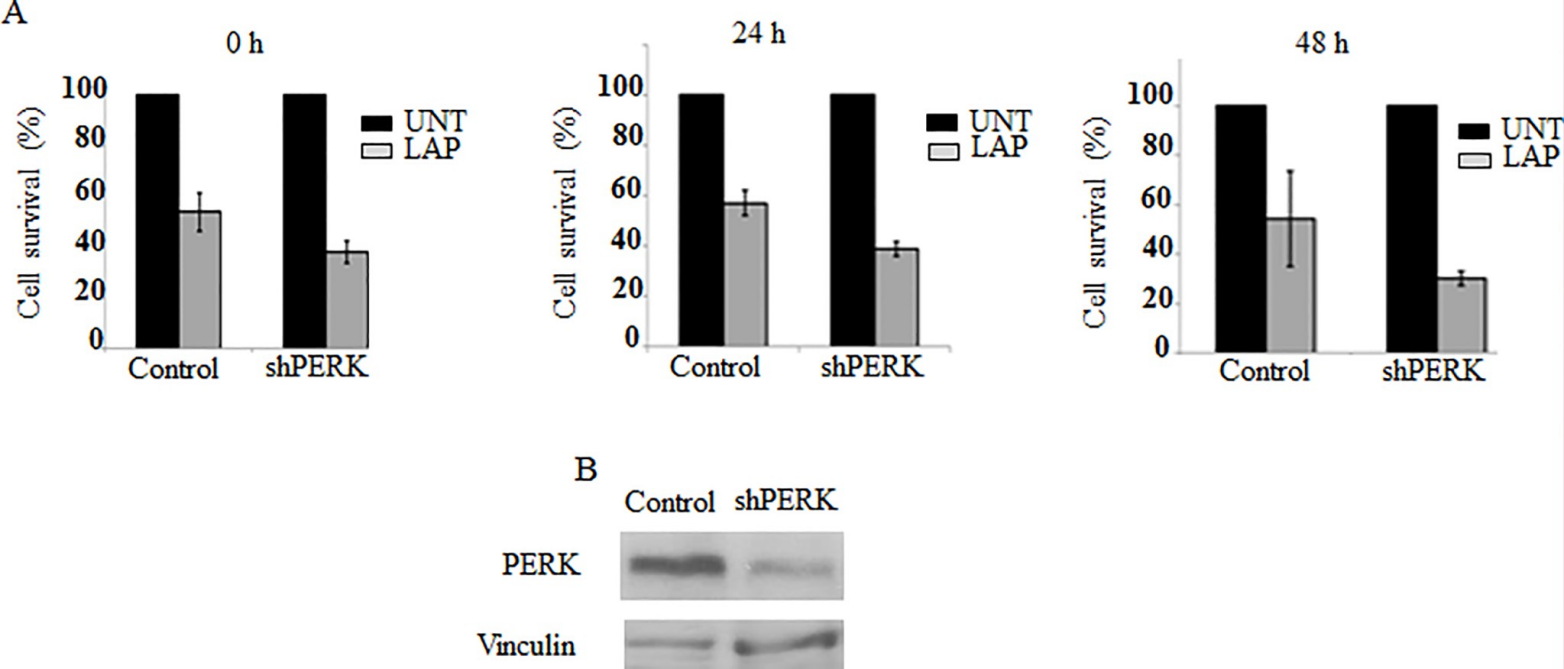
PERK depletion sensitizes T47D to LAP. (A) The indicated shRNA-expressing T47D were treated with LAP (20 μM) for twenty-four hours, washed with PBS and MTT solvent was added at the indicated time points for an additional two hours. The absorbance was monitored at 560 nm. Cell viability was calculated as the percentage of the absorbance values of LAP-treated cells relative to the absorbance values corresponding to untreated cells. Values are presented as the mean percentage +/- SD. (B) Depletion of PERK in shRNA PERK-expressing T47D was validated by western blot analysis using anti-PERK antibodies and quantified as above.

Given the role of SG formation in cell resistance to stress, we sought to determine if the activation of SG formation pathway participates to T47D resistance to Lap, using MTT assay. Control experiments show that Lap treatment induces cell death in ~50% of T47D (Fig 4). Depletion of PERK further sensitises T47D to Lap treatment leading to ~63% of cell death (Fig 4), supporting the assumption that PERK and associated SG formation contribute to the resistance of cancer cells to Lap.

## Discussion

In this study, we identified Lap, a chemotherapeutic drug used to treat breast cancer, as a new SG inducer (Fig 1). This formation of SG correlates with an inhibition of general translation that is triggered by the activation of PERK and downstream eIF2α phosphorylation (Figs 2 and 3 and S4 Data). Both ISRIB and PERK depletion experiments support a major role of PERK-eIF2α phosphorylation, though we do not exclude a contribution of additional pathways including the non-canonical 4EBP1 pathway [35] or mTORC1 [22] pathway. Finally, the activation of PERK-SG formation pathway contributes to the resistance of the breast T47D cancer cells to death induced by Lap treatment.

While the formation of SG induced by either environmental (e.g., low and high temperature, radiation, oxygen and nutrient deprivation, arsenite treatment and genotoxic drugs not relevant to cancer), or pathologic (e.g., viral infection) has been well documented, studies investigating their formation triggered by chemotherapeutics are still limited. So far, four chemotherapeutics has been characterised as potent SG inducers in cancer cells. Our previous studies have identified two of these, namely bortezomib and sorafenib. The formation of SG that occurs during treatment with either drug is not due to the inactivation of their targets per se, but involves the activation of a cellular stress response characterised by phosphorylation of eIF2α through upstream specific kinases. For example, in addition of inhibiting its proteasome target, bortezomib generates an oxidative stress that triggers SG formation by activating HRI-PeIF2α pathway [22,30]. Treatment with sorafenib, a RAF Tyr kinases inhibitor activates an additional ER stress pathway that induces SG in a PERK-PeIF2α dependent manner [25]. Our finding that Lap, a ErbB1/2 Tyr kinases inhibitor, also triggers SG formation by activating PERK, raises the possibility that targeting Tyr kinases family may engender the activation of a common ER stress activating PERK and SG formation. However, PERK-PeIF2α pathway was also recently identified as a driver of SG formation in cancer cells treated with specific members of the anti-mitotic microtubule destabilising Vinca alkaloids chemotherapeutic family, although with different efficiencies [36]. Formation of SG induced by the 5-Fluorouracil (5-FU) also involves eIF2α phosphorylation, though the activating stress kinase remains to be established [37]. While 5-FU is known as a DNA-incorporating drug, it is its incorporation into RNA that seems the primary driver of eIF2α phosphorylation and downstream SG formation, predicting that incorporation of specific chemotherapeutics into RNA, and not at their natural target DNA, may constitute a potential mechanism of SG induction. How the incorporation of 5-FU into RNA activates phosphorylation of eIF2α pathway inducing SG formation is not known. Nevertheless, these studies together with ours showing that Lap induces SG formation by activating eIF2α phosphorylating pathway confirm a major contribution of this pathway in SG formation during treatment with different class of chemotherapeutics. Future studies will help to determine which eIF2α phosphorylating kinase is activated to trigger SG formation upon treatment with each specific class of chemotherapeutics.

Our time course experiments of SG formation show a correlation between SG formation and T47D resistance to Lap treatment. We show that the maximal SG formation (~70%) lasted over 6h, before decreasing to 25% after 24 h of Lap treatment. These results indicate that while in the majority of cells, SG are transient, they remained stable in a significant fraction of cells during treatment, which may contribute to the observed resistance of T47D to Lap treatment. This is consistent with our PERK depletion experiments showing that targeting PERK-SG pathway further sensitizes T47D to Lap and supporting a potential role of PERK-SG formation pathway in resistance of cancer cells to drug treatment. While these results are in line with the role of SG in antagonising cell death [38], they are consistent with the emerging role of PERK in the promotion of tumor growth and angiogenesis [39–41]. Although the contribution of SG in PERK-mediated tumor growth and angiogenesis remained to be established, our study described here suggests the formation of SG as a potential downstream effector of PERK in Lap resistance, an assumption that requires future *in vivo* studies using patient-derived xenografts and breast biopsies of cancer patients, to be validated. While SG impact cell death by sequestering signaling death molecules [14,15], we [16,22] and others [17] have implicated SG in cell resistance to stress-mediated cell death in part by preventing degradation of mRNAs encoding survival functions. Future studies assessing transcriptomes differences between cell lines-forming and–lacking SG (e.g., between mock- and PERK-depleted T47D) should reveal specific mRNAs whose amounts, though not affected by either Lap treatment or PERK depletion, are significantly downregulated in SG-deficient cells treated with Lap, potentially at their stability level. Identifying SG-Lap mRNA targets should also reveal specific mRNAs whose regulation by SG impact cell resistance to LAP, providing specific mechanisms of cancer chemoresistance. Nevertheless, our *in vitro* study supports the possibility that induction of SG formation by chemotherapeutics constitutes a general mechanism, potentially involved in chemoresistance.

## Supporting information

S1 Data LAP induces SG in MCF-7 but not in MDA-MB-231. MCF-7 and MDA-MB-231 were treated with LAP (20 μM) for two hours. Cells were fixed, permeabilized and processed for immunofluorescence using antibodies against the SG markers FMRP and FXR1. DAPI is used as a marker for nuclei.
(TIF)

S2 Data Assessment of P-bodies formation in LAP-treated cells. U2OS were treated with LAP (20 μM) for two hours, fixed and P-bodies were visualised by immunofluorescence using either anti-Dcp1a or anti-LSM14A antibodies. Anti-FMRP antibodies are used to decorate SG.
(TIF)

S3 Data LAP treatment reduces phosphorylation of 4EBP1. T47D were treated with LAP (20 μM) for two hours. Protein extracts were prepared and their content analysed by western blot using antibodies specific to the indicated proteins. Tubulin (Tub) serves as loading control.
(TIF)

S4 Data LAP induces SG in wild type MEFs but not in mutant versions that do not phosphorylate eIF2α the indicated MEFs were treated with LAP (20 αM) for two hours, fixed and SG were visualised by immunofluorescence using anti-FMRP and -FXR1 antibodies. DAPI is used as a marker for nuclei.
(TIF)

S1 Fig (TIF)

## References

[1] Protter D. S. & Parker R. Principles and Properties of Stress Granules. *Trends Cell Biol* 26, 668–679, 10.1016/j.tcb.2016.05.004 (2016). 27289443 PMC4993645

[2] Van Treeck B. & Parker R. Emerging Roles for Intermolecular RNA-RNA Interactions in RNP Assemblies. *Cell* 174, 791–802, 10.1016/j.cell.2018.07.023 (2018). 30096311 PMC6200146

[3] Van Treeck B. et al RNA self-assembly contributes to stress granule formation and defining the stress granule transcriptome. *Proc Natl Acad Sci U S A* 115, 2734–2739, 10.1073/pnas.1800038115 (2018). 29483269 PMC5856561

[4] Kedersha N., Ivanov P. & Anderson P. Stress granules and cell signaling: more than just a passing phase? *Trends Biochem Sci* 38, 494–506, 10.1016/j.tibs.2013.07.004 (2013). 24029419 PMC3832949

[5] Panas M. D., Ivanov P. & Anderson P. Mechanistic insights into mammalian stress granule dynamics. *J Cell Biol* 215, 313–323, 10.1083/jcb.201609081 (2016). 27821493 PMC5100297

[6] Donnelly N., Gorman A. M., Gupta S. & Samali A. The eIF2alpha kinases: their structures and functions. *Cell Mol Life Sci* 70, 3493–3511, 10.1007/s00018-012-1252-6 (2013). 23354059 PMC11113696

[7] Wek R. C., Jiang H. Y. & Anthony T. G. Coping with stress: eIF2 kinases and translational control. *Biochem Soc Trans* 34, 7–11 (2006). 10.1042/BST20060007 16246168

[8] Castilho B. A. et al Keeping the eIF2 alpha kinase Gcn2 in check. *Biochim Biophys Acta* 1843, 1948–1968, 10.1016/j.bbamcr.2014.04.006 (2014). 24732012

[9] Taylor S. S., Haste N. M. & Ghosh G. PKR and eIF2alpha: integration of kinase dimerization, activation, and substrate docking. *Cell* 122, 823–825, 10.1016/j.cell.2005.09.007 (2005). 16179248

[10] Joncas F. H., Adjibade P. & Mazroui R. [Role of HRI in apoptosis resistance]. *Med Sci (Paris)* 30, 882–888, 10.1051/medsci/20143010015 (2014). 25311023

[11] Wek R. C. & Cavener D. R. Translational control and the unfolded protein response. *Antioxid Redox Signal* 9, 2357–2371, 10.1089/ars.2007.1764 (2007). 17760508

[12] Kedersha N. & Anderson P. Stress granules: sites of mRNA triage that regulate mRNA stability and translatability. *Biochem Soc Trans* 30, 963–969 (2002). 10.1042/bst0300963 12440955

[13] Van Treeck B. & Parker R. Principles of Stress Granules Revealed by Imaging Approaches. *Cold Spring Harb Perspect Biol* 11, 10.1101/cshperspect.a033068 (2019). 30709880 PMC6360856

[14] Arimoto K., Fukuda H., Imajoh-Ohmi S., Saito H. & Takekawa M. Formation of stress granules inhibits apoptosis by suppressing stress-responsive MAPK pathways. *Nat Cell Biol* (2008). 10.1038/ncb1791 18836437

[15] Thedieck K. et al Inhibition of mTORC1 by Astrin and Stress Granules Prevents Apoptosis in Cancer Cells. *Cell* 154, 859–874, 10.1016/j.cell.2013.07.031 (2013). 23953116

[16] Gareau C. et al p21(WAF1/CIP1) upregulation through the stress granule-associated protein CUGBP1 confers resistance to bortezomib-mediated apoptosis. *PLoS One* 6, e20254, 10.1371/journal.pone.0020254 (2011). 21637851 PMC3102688

[17] Moeller B. J., Cao Y., Li C. Y. & Dewhirst M. W. Radiation activates HIF-1 to regulate vascular radiosensitivity in tumors: role of reoxygenation, free radicals, and stress granules. *Cancer Cell* 5, 429–441 (2004). 10.1016/s1535-6108(04)00115-1 15144951

[18] Chu I., Blackwell K., Chen S. & Slingerland J. The dual ErbB1/ErbB2 inhibitor, lapatinib (GW572016), cooperates with tamoxifen to inhibit both cell proliferation- and estrogen-dependent gene expression in antiestrogen-resistant breast cancer. *Cancer Res* 65, 18–25 (2005). 15665275

[19] MacFarlane R. J. & Gelmon K. A. Lapatinib for breast cancer: a review of the current literature. *Expert Opin Drug Saf* 10, 109–121, 10.1517/14740338.2011.533168 (2011). 21091041

[20] D’Amato V. et al Mechanisms of lapatinib resistance in HER2-driven breast cancer. *Cancer Treat Rev* 41, 877–883, 10.1016/j.ctrv.2015.08.001 (2015). 26276735

[21] Shi H., Zhang W., Zhi Q. & Jiang M. Lapatinib resistance in HER2+ cancers: latest findings and new concepts on molecular mechanisms. *Tumour Biol*, 10.1007/s13277-016-5467-2 (2016). 27726101

[22] Fournier M. J. et al Inactivation of the mTORC1-eukaryotic translation initiation factor 4E pathway alters stress granule formation. *Mol Cell Biol* 33, 2285–2301, 10.1128/MCB.01517-12 (2013). 23547259 PMC3648080

[23] Mazroui R., Di Marco S., Kaufman R. J. & Gallouzi I. E. Inhibition of the ubiquitin-proteasome system induces stress granule formation. *Mol Biol Cell* 18, 2603–2618 (2007). 10.1091/mbc.E06-12-1079 17475769 PMC1924830

[24] Mazroui R. et al Inhibition of ribosome recruitment induces stress granule formation independently of eukaryotic initiation factor 2alpha phosphorylation. *Mol Biol Cell* 17, 4212–4219 (2006). 10.1091/mbc.E06-04-0318 16870703 PMC1635342

[25] Adjibade P. et al Sorafenib, a multikinase inhibitor, induces formation of stress granules in hepatocarcinoma cells. *Oncotarget*, 10.18632/oncotarget.5980 (2015). 26556863 PMC4791277

[26] Huang H., Weng H. & Chen J. The Biogenesis and Precise Control of RNA m(6)A Methylation. *Trends Genet* 36, 44–52, 10.1016/j.tig.2019.10.011 (2020). 31810533 PMC6925345

[27] Anders M. et al Dynamic m(6)A methylation facilitates mRNA triaging to stress granules. *Life Sci Alliance* 1, e201800113, 10.26508/lsa.201800113 (2018). 30456371 PMC6238392

[28] Adjibade P. & Mazroui R. Control of mRNA turnover: implication of cytoplasmic RNA granules. *Semin Cell Dev Biol* 34, 15–23, 10.1016/j.semcdb.2014.05.013 (2014). 24946962

[29] Kedersha N. et al Stress granules and processing bodies are dynamically linked sites of mRNP remodeling. *J Cell Biol* 169, 871–884 (2005). 10.1083/jcb.200502088 15967811 PMC2171635

[30] Fournier M. J., Gareau C. & Mazroui R. The chemotherapeutic agent bortezomib induces the formation of stress granules. *Cancer Cell Int* 10, 12, 10.1186/1475-2867-10-12 (2010). 20429927 PMC2873330

[31] Kedersha N. et al Dynamic shuttling of TIA-1 accompanies the recruitment of mRNA to mammalian stress granules. *J Cell Biol* 151, 1257–1268 (2000). 10.1083/jcb.151.6.1257 11121440 PMC2190599

[32] Mazroui R. et al Trapping of messenger RNA by Fragile X Mental Retardation protein into cytoplasmic granules induces translation repression. *Hum Mol Genet* 11, 3007–3017 (2002). 10.1093/hmg/11.24.3007 12417522

[33] Cruickshanks N. et al Lapatinib and obatoclax kill breast cancer cells through reactive oxygen species-dependent endoplasmic reticulum stress. *Mol Pharmacol* 82, 1217–1229, 10.1124/mol.112.081539 (2012). 22989520 PMC3502625

[34] Sidrauski C., McGeachy A. M., Ingolia N. T. & Walter P. The small molecule ISRIB reverses the effects of eIF2alpha phosphorylation on translation and stress granule assembly. *Elife* 4, 10.7554/eLife.05033 (2015). 25719440 PMC4341466

[35] Fujimura K., Sasaki A. T. & Anderson P. Selenite targets eIF4E-binding protein-1 to inhibit translation initiation and induce the assembly of non-canonical stress granules. *Nucleic Acids Res*, 10.1093/nar/gks566 (2012). 22718973 PMC3439927

[36] Szaflarski W. et al Vinca alkaloid drugs promote stress-induced translational repression and stress granule formation. *Oncotarget* 7, 30307–30322, 10.18632/oncotarget.8728 (2016). 27083003 PMC5058682

[37] Kaehler C., Isensee J., Hucho T., Lehrach H. & Krobitsch S. 5-Fluorouracil affects assembly of stress granules based on RNA incorporation. *Nucleic Acids Res* 42, 6436–6447, 10.1093/nar/gku264 (2014). 24728989 PMC4041438

[38] Gao X. et al Stress granule: A promising target for cancer treatment. *Br J Pharmacol* 176, 4421–4433, 10.1111/bph.14790 (2019). 31301065 PMC6932939

[39] Fels D. R. & Koumenis C. The PERK/eIF2alpha/ATF4 module of the UPR in hypoxia resistance and tumor growth. *Cancer Biol Ther* 5, 723–728 (2006). 10.4161/cbt.5.7.2967 16861899

[40] Karali E. et al VEGF Signals through ATF6 and PERK to promote endothelial cell survival and angiogenesis in the absence of ER stress. *Mol Cell* 54, 559–572, 10.1016/j.molcel.2014.03.022 (2014). 24746698

[41] Koromilas A. E. Roles of the translation initiation factor eIF2alpha serine 51 phosphorylation in cancer formation and treatment. *Biochim Biophys Acta*, 10.1016/j.bbagrm.2014.12.007 (2014). 25497381

