# Supplementary figures and images for: Treatment of cancer cells with Lapatinib negatively regulates general translation and induces stress granules formation
Source: PLoS One. 2020 May 4;15(5):e0231894. doi: 10.1371/journal.pone.0231894 (PMC7197775; doi:10.1371/journal.pone.0231894)

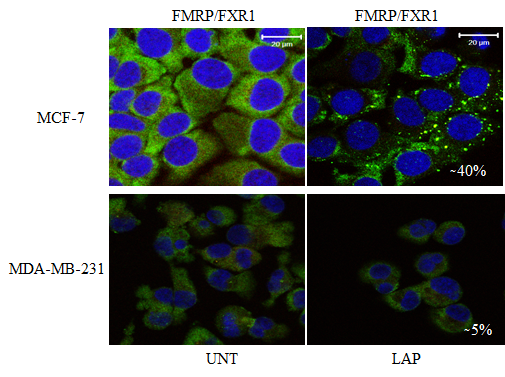

Supplement: S1 Data — MCF-7 and MDA-MB-231 were treated with LAP (20 μM) for two hours. Cells were fixed, permeabilized and processed for immunofluorescence using antibodies against the SG markers FMRP and FXR1. DAPI is used as a marker for nuclei. (TIF) [file pone.0231894.s001.tif]

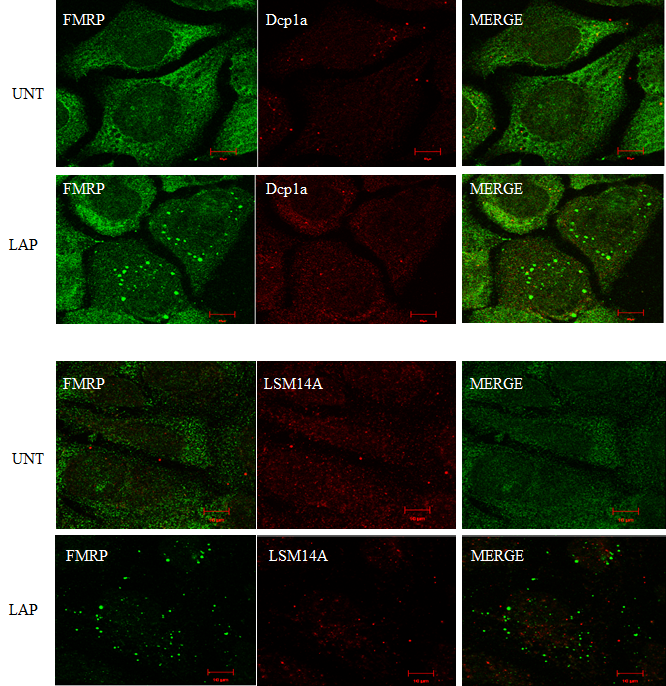

Supplement: S2 Data — U2OS were treated with LAP (20 μM) for two hours, fixed and P-bodies were visualised by immunofluorescence using either anti-Dcp1a or anti-LSM14A antibodies. Anti-FMRP antibodies are used to decorate SG. (TIF) [file pone.0231894.s002.tif]

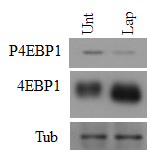

Supplement: S3 Data — T47D were treated with LAP (20 μM) for two hours. Protein extracts were prepared and their content analysed by western blot using antibodies specific to the indicated proteins. Tubulin (Tub) serves as loading control. (TIF) [file pone.0231894.s003.tif]

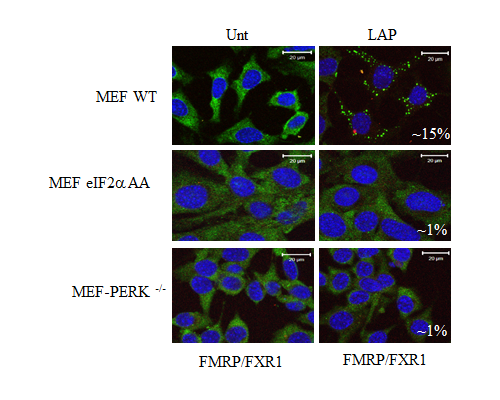

Supplement: S4 Data — DAPI is used as a marker for nuclei. (TIF) [file pone.0231894.s004.tif]

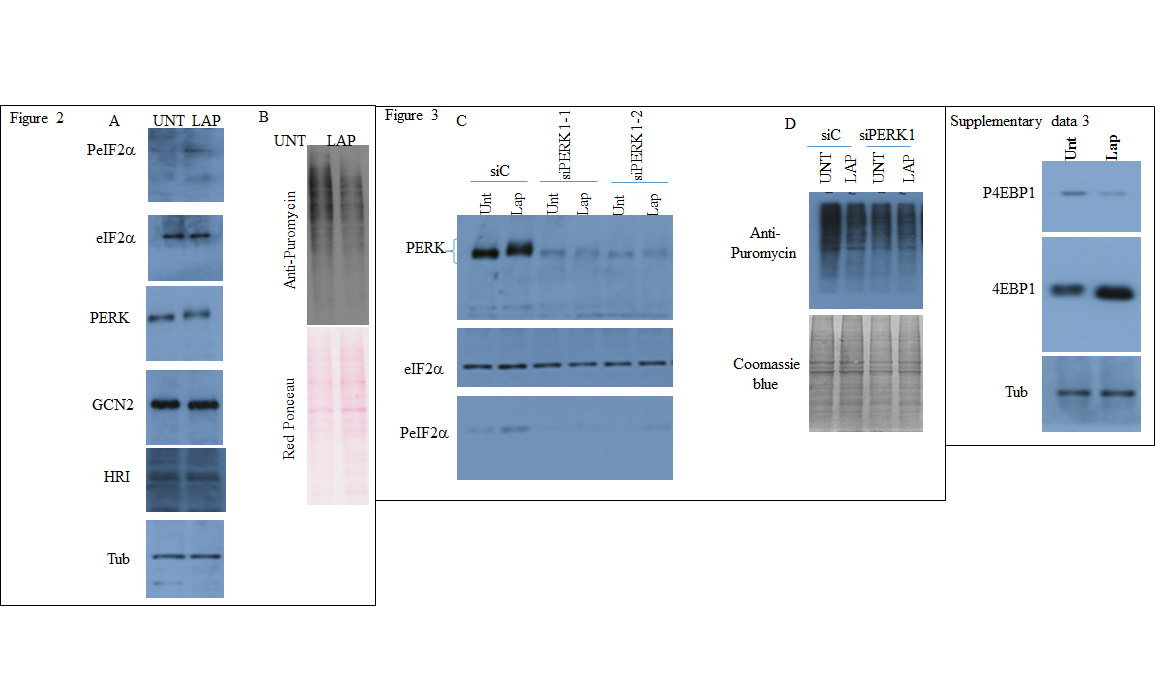

Supplement: S1 Fig — (TIF) [file pone.0231894.s005.tif]
